# Effect of oral cannabis administration on the fat depots of obese and streptozotocin‐induced diabetic rats

**DOI:** 10.1002/ptr.7694

**Published:** 2022-11-27

**Authors:** Sonaal Ramlugon, Ruby‐Ann Levendal, Carminita L. Frost

**Affiliations:** ^1^ Department of Biochemistry and Microbiology Nelson Mandela University Port Elizabeth South Africa

**Keywords:** beigeing, cannabis, intramuscular, mitochondria, peritoneal, UCP1

## Abstract

The prevalence of obesity and insulin‐resistance is on the rise, globally. Cannabis have been shown to have anti‐diabetic/obesity properties, however, the effect mediated at various fat depots remains to be clarified. The aim of this study was to (1) investigate the anti‐diabetic property of an oral cannabis administration in an obese and streptozotocin‐induced diabetic rat model and (2) to determine and compare the effect mediated at the peritoneal and intramuscular fat level. Cannabis concentration of 1.25 mg/kg body weight (relative to THC content) was effective in reversing insulin‐resistance in the rat model, unlike the other higher cannabinoid concentrations. At the peritoneal fat level, gene expression of fat beigeing markers, namely Cidea and UCP1, were significantly increased compared to the untreated control. At the intramuscular fat level, on the other hand, CE1.25 treatment did not promote fat beigeing but instead significantly increased mitochondrial activity, relative to the untreated control. Therefore, these findings indicate that the mechanism of action of oral cannabis administration, where glucose and lipid homeostasis is restored, is not only dependent on the dosage but also on the type of fat depot investigated.

**Highlights**

- CE1.25 Improved fasting glucose levels and potentially reversed insulin‐resistance.
- Increased expression of beige markers in peritoneal fat.
- Increased mitochondrial activity in intramuscular fat.
- Effect observed is dependent on dosage administered and type of fat deposit.

## 1 INTRODUCTION

Obesity is tightly linked to the development of metabolic syndromes such as insulin‐resistance, blood lipid disorders, hypertension, and inflammation (Arhire et al., 2019; Després & Lemieux, 2006; Kim et al., 2021). One of the most powerful therapeutic tools to combat obesity is through induction of beige adipocytes within the white adipose tissue (WAT) of the body (Stine et al., 2016; Thyagarajan & Foster, 2017; Wang & Seale, 2016), often through activation of the beta‐adrenergic receptor signaling pathway. Unfortunately, such a strategy is commonly associated with serious side‐effects such as hypertension and heart diseases (Pan et al., 2020). Therefore, a more effective and safer treatment option is required. Fat depots play an important role in the body. They act as endocrine organs (Coelho et al., 2013; Galic et al., 2010; Murawska‐Ciałowicz, 2017) where they regulate glucose, lipid, and energy metabolism (Kojta et al., 2020; Song et al., 2017); provide insulation and protect non‐adipose organs such as the liver, heart, muscle, pancreas, and kidney against lipotoxicity. The ability of adipocytes to expand and store excess lipids is limited, and often when storage capacity is exceeded, there is lipid overflow to other peripheral organs ultimately leading to the occurrence of metabolic disorders (Slawik & Vidal‐Puig, 2006; Virtue & Vidal‐Puig, 2010). By promoting fat beigeing and or fat oxidation in the main fat depots, surrounding vital organs such as the peritoneal fat, would help in reducing lipid overflow and possibly reverse the obesity state of the individual or even maintain metabolic healthy obesity. Metabolic healthy obesity is not associated with the development of metabolic syndrome such as insulin resistance as glucose/lipid metabolism and insulin sensitivity are preserved (Ahmad & Zawatia, 2021; Blüher, 2020; Tsatsoulis & Paschou, 2020). It has been found to be strongly dependent on the type and location of fat depots (Bala et al., 2016; Gómez‐Zorita et al., 2021; Teixeira et al., 2015), for example, an increase in peritoneal fat and subcutaneous fat depots is linked to an increased and decreased risk of metabolic syndrome, respectively. Therefore, it would be of great interest to researchers to design or discover a drug, that can optimize fat distribution and metabolism in the body. This would help tackle the worldwide occurrence of obesity, obesity‐linked insulin resistance and potentially other metabolic disorders such as cardiovascular diseases. Cannabis has been extensively known for its medicinal and recreational use. Several studies have demonstrated its anti‐diabetic and anti‐obesity properties (Levendal et al., 2012; Ramlugon et al., 2018). However, the mechanism of action on fat depots is unknown. Whether cannabis and/or phytocannabinoids increase metabolic activity or promotes beigeing in adipose tissue remains to be clarified. The aim of this study is therefore to determine (1) the *in vivo* effects mediated by oral cannabis administration, (2) the role of dosage administered, (3) morphological changes mediated at various fat depots and (4) the mechanism of action, focusing mainly at peritoneal and intramuscular fat level, in a rat model mimicking an obese and insulin‐resistant state.

## 2 MATERIALS AND METHODS

### 2.1 Cannabis extraction and quantification

A chloroform‐based extraction was performed according to a modified protocol as previously described (Levendal et al., 2012; Ramlugon et al., 2018) and the extract was quantified using Reverse Phased High‐Performance Liquid Chromatography (RP‐HPLC). Briefly, whole plant (*Cannabis sativa* L.) material was finely chopped (Data supplied as Appendix S1). Analytical grade chloroform was added to cover the plant material and the mixture was kept overnight at 4°C in the dark. The supernatant was then removed, fresh chloroform was added to the plant material and left for 4 h. The process was repeated one more time and the three fractions were combined and filtered to remove any plant debris, using firstly Whatman No.1 filter paper and subsequently 0.45 μm Millex Millipore filters. Nitrogen gas was used to evaporate the chloroform at 4°C in the dark. The remaining resin was dissolved in minimum volume of methanol. For quantification, the sample was eluted using the Restek Raptor™ ARC‐18 column (4.6 × 150 mm) and elution buffers of 0.1% formic acid in acetonitrile and 0.1% formic acid in water in a ratio of 75:25, respectively, at a flow rate of 1.5 ml/min. Commercial cannabinoids namely CBDV, CBG, CBN, CBD, and THC (1 mg/ml) purchased from Sigma, Leco and Industrial Analytical, were used to construct respective standards curves. The amount of THC: CBN: CBD: CBG: CBDV in the extract was found to be in a ratio of 5:2.5:1:0.1:0.7, respectively. After HPLC quantification, methanol was evaporated using nitrogen gas and the remaining resin was dissolved in Extra Virgin Olive Oil (South Africa) and administered orally (by swallowing and according to IACUC compliance) to the experimental rats. This enteral route of administration helped to minimize discomfort and stress on the rats as opposed to injections.

### 2.2 Experimental dessign (Obese and STZ‐induced diabetic rat model)

Animal Ethics Clearance (A16‐SCI‐BCM‐001) was obtained from Research Ethics Committee (Animals) of Nelson Mandela university of Port Elizabeth. Male Wistar rats (*Rattus norvegicus*) of 7 weeks old, weighing approximately 166 ± 5 g were purchased from the Biomedical Resource Unit of University of KwaZulu Natal (Durban – South Africa). The rats were randomly assigned to six groups (seven rats each) namely Lean, STZ, CE1.25, CE2.5, CE5.0, and MET. The rats were kept in an air‐conditioned room (22 ± 3°C) under a 12‐h light/dark cycle. During the first week of acclimatization all the rats were fed the EPOL Mice Cubes (EPOL) with water available *ad libitum*. Afterwards, the Lean group was maintained on the EPOL diet whilst all the other groups were allocated a HFD diet (HFD ‐ D12492 from Research Diet USA) for the entire duration of the experiment (18 weeks). The percentage kilocalorie content was 22% protein, 7% fat, and 71% carbohydrate for the EPOL diet and 20% protein, 60% fat, and 20% carbohydrates for the HFD diet. All the rats were weighed every week. After 10 weeks on diet, rats of the STZ, CE1.25, CE2.5, CE5.0, and MET groups were intraperitoneally injected with 30 mg/kg body weight streptozotocin (STZ) to induce insulin resistance whereas the Lean group were intraperitoneally injected with the citrate buffer pH 4.5 (vehicle control). Intraperitoneal glucose tolerance tests (IPGTT) and intraperitoneal insulin tolerance tests (IPITT) was conducted before and after treatment ended. Rats in the CE1.25, CE2.5, CE5.0, and MET groups were then orally treated, every alternate day for further 8 weeks, with cannabis extract concentration of 1.25, 2.5, 5.0 mg/kg body weight (relative to THC content of the extract), and 50 mg/kg metformin, respectively, whilst the control groups namely Lean and STZ were given Extra Virgin Olive Oil (South Africa). After 8 weeks on treatment, the rats were sacrificed, and various fat depots namely peritoneal, intramuscular, mesenteric, epididymal, sternum, pancreatic, and brown fat (BAT) were harvested.

### 2.3 IPGTT (Intraperitoneal glucose tolerance test), IPITT (Intraperitoneal insulin tolerance test), Cholesterol, and Triacylglycerides measurements

IPGTT and IPITT were conducted before and after treatment. Briefly, the rats were fasted overnight for a period of 13–14 h and were weighed just before the start of the experiment. For the IPGTT experiment, the rats were intraperitoneally injected with a glucose solution at a dosage of 2 g/kg body weight (Levendal et al., 2012; Matheka et al., 2011). Blood samples were taken from the tails (puncture of tail vein) before an intraperitoneal glucose injection and after 30, 60, 90, and 120 min after the glucose bolus injection. Accu‐chek strips (Roche Diagnostics) were used to measure the blood glucose concentrations of the rats. In the case of the IPITT experiment, the procedures were similar except the rats were injected intraperitoneally with insulin (instead of glucose) at a dosage of 0.5 U/kg body weight (Guo et al., 2018). Total cholesterol and triacylglycerides levels of fasted rats were measured on days when IPGTT experiments were conducted and Accutrend Plus strips (Roche) were used. Fasting plasma insulin levels were measured using the Rat Insulin Eliza kit (Thermo Fisher Scientific) according to the manufacturer's protocol. HOMA2‐%B, HOMA2‐%S, and HOMA2‐IR were calculated using the online HOMA2 calculator (https://www.dtu.ox.ac.uk/homacalculator).

### 2.4 Mitochondrial to genomic DNA ratio (MT:18S)

DNA was extracted from various fat depots namely peritoneal, intramuscular, mesenteric, epididymal, pancreatic, sternum, and brown adipose tissue using the Qiagen DNeasy Blood and Tissue kit, according to the manufacturer's guidelines and PCR was conducted using Taq2x Master Mix (BioLabs). Mitochondrial primer (forward 5′–3′: GCAGCCACAGGAAAATCCG, reverse 5′–3′: GTAGGGCAGAGACGGGAGTTG) and genomic (18S ribosomal) primer (forward 5′–3′: GTTGGTTTTCGGAACTGAGGC, reverse 5′–3′: GTCGGCATCGTTTATGGTCG) were used for quantification of MT:18S. The PCR conditions were as follows: (Step 1) Initial denaturation at 95°C for 2 min; (Step 2–25 cycles) denaturation at 95°C for 1 min, annealing at 60.8°C for 15 s, and elongation at 68°C for 30 s; and (Step 3) final elongation at 68°C for 5 min. The PCR products were ran at 100 V for 45 min on a 2.5% agarose gel and visualized using the Image DOCK system. The image J software was used to determine the relative intensity of the bands and calculate the MT:18S.

### 2.5 Protein extraction and quantification

Protein extraction and quantification was conducted as previously described (Lindinger et al., 2015). Briefly, adipose tissue was homogenized in homogenization buffer containing 200 mM mannitol, 50 mM sucrose, 1 mM EDTA, and 20 mM HEPES, pH 7.4. The homogenate was then centrifuged at 700 *g* for 11 min to collect the cell debris and the supernatant was then centrifuged at 7000 *g* for 19 min to pellet the mitochondrial fraction. Bicinchoninic acid (BCA) assay was conducted for protein quantification.

### 2.6 Citrate synthase activity

Citrate synthase enzymatic activity was measured using a modified protocol described by (Christe et al., 2013). Briefly, the reaction mixture contained 0.25% Triton‐X‐100, 0.31 mM Acetyl‐CoA, 0.1 mM DTNB (5,5‐dithio‐bis, 2‐nitrobenzoic acid), and 0.5 mM Oxaloacetate. The reaction volume was adjusted accordingly to obtain a final volume of 200 μl. The formation of thionitrobenzoic acid (TNB) from DTNB was measured spectrophotometrically at 412 nm.

### 2.7 CPT1 activity

CPT1 enzymatic activity was measured according to a previous protocol (Shimoda et al., 2006). Briefly, 58 mM Tris buffer (containing 1.25 mM EDTA, 0.1%Triton‐X‐100 and 0.25 mM DTNB), 37.5 μM palmitoyl CoA, and 1.25 mM l‐carnitine was used to set up the reaction. The formation of TNB from DTNB was monitored spectrophotometrically at 412 nm.

### 2.8 Histology

Fat tissues namely peritoneal, intramuscular, mesenteric, epididymal, pancreatic, sternum, and BAT stored in formaldehyde at −20°C were used for histology experiments. The samples were processed using the automatic Leica TP1020 tissue processor and then embedded in paraffin wax. Sections of 7uM thickness was stained with Hematoxylin and Eosin (Cardiff et al., 2014). The Olympus Stream image analysis software (Version 2.4.2) was used to measure the area of 700 random adipocytes for each group. Heatmaps were generated using the shinymap software (Khomtchouk et al., 2017).

### 2.9 qPCR

RNA extraction (peritoneal, intramuscular and BAT) was performed using the RNeasy Lipid Tissue Mini kit from Qiagen and the concentration and quality of RNA extracted was determined using the RNA Nano Chip (Bioanalyzer). Samples with RIN values higher than 6 was converted to cDNA using the Quantitect Reverse Transcription kit (Qiagen). qPCR was conducted using the Luna Universal qPCR mastermix (BioLabs) and CFX Connect Real‐Time PCR Detection System. Qbase^+^ software (Bio‐Rad) was used to analyze the data and normalize to housekeeping genes. GeNorm analysis was performed to determine the stability of the housekeeping genes (M value <1.5) across all the experimental conditions. Based on the M value generated after GeNorm analysis, data for peritoneal fat was normalized against Cyclophilin A (Forward 5′–3′: TCACCATCCCGACTGTGGA, Reverse primer 5′–3′: AAATGCCCGCAAGTCAAAGA) and 18S (Forward 5′–3′: GGAGAGGGAGCCTGAGAAAC, Reverse 3'–5′: GGAGAGGGAGCCTGAGAAAC), intramuscular data against Cyclophilin A and UBC (Forward 3′–5′: GACAGGCAAGACCATCACTC, Reverse 5′–3′: CCAAGAACAAGCACAAGAAGG) and BAT data against 18S and UBC. The target genes investigated include Uncoupling Protein 1 (UCP1; Forward 5′–3′: TGGCATCCAGAGGCAAATC, Reverse 5′–3′: GCATTGTAGGTCCCAGTGTAG), Cell‐Death Inducing DFFA Effector A (Cidea; Forward 5′–3′: GTTTATGCGGGCGCTTATG, Reverse 5′–3′: TTCTCTTGCGGGAATCCTG), Perilipin (Forward 5′–3′: ACACTCTTTCTCGACACACC, Reverse 5′–3′: CTGGTCTTCATGGTTCTCAT), Hormone‐Sensitive Lipase (HSL; Forward 5′–3′: AGGACACCTTGGCTTGAGCG, Reverse 5′–3′: TGCCCAGGAGTGTGTCTGAG), and Transcription factor A Mitochondrial (TFAM; Forward 5′–3′: GGGATTGGGCACAAGAAG, Reverse 5′–3′: GCATTCAGTGGGCAGAAG).

## 3 RESULTS AND DISCUSSION

Cannabis is widely known for its anti‐diabetic properties (Comelli et al., 2009; Nuutinen, 2018; Ramlugon et al., 2018; Weiss et al., 2006). However, from this study, it has been found that the dosage of cannabis plays a crucial role in the effect mediated and is dependent on the type of fat depot investigated. Cannabinoids often exhibit a biphasic nature (Mechoulam & Parker, 2013) where a low dosage can have an opposing effect in comparison to a higher dosage, for example, a low and high THC dosage have been shown to have an appetite promoting and reducing effect respectively (Horn et al., 2018). Furthermore, a low dosage of THC has been shown to promote hyperthermia in rats whilst a higher dosage induced hypothermia (Taylor & Fennessy, 1977). Another study involving zebrafish, has shown that a low dosage of THC promotes longevity, fertility, and reduces inflammatory markers, whilst a higher dosage does the opposite (Pandelides et al., 2020). Therefore, it is crucial to determine the optimum concentration for specific medicinal use to avoid unwanted or opposing effects.

### 3.1 The significance of optimum cannabis dosage in mediating anti‐diabetic effects in STZ‐induced diabetic rat model

**FIGURE 1:**
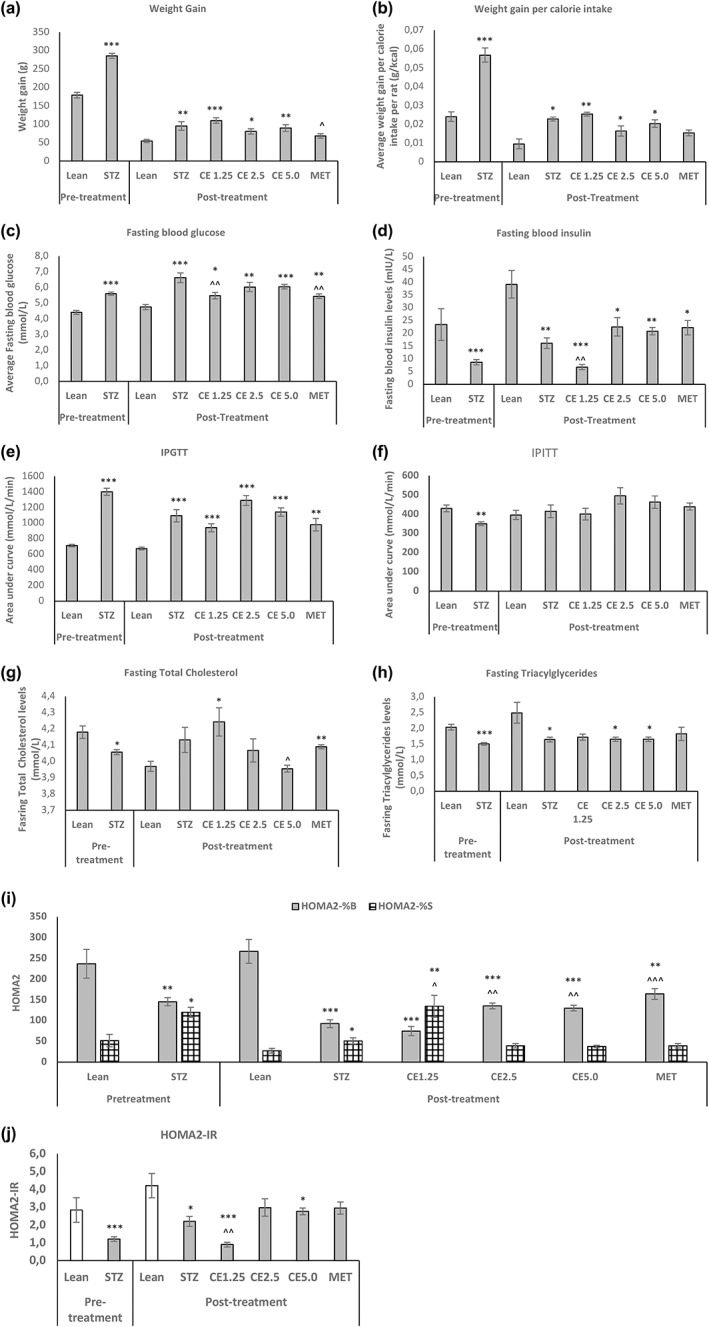
The *in vivo* parameters of the rats before and after treatment. The effect of oral cannabis administration on (a) weight gain, (b) weight gain per calorie intake, (c) fasting glucose, (d) fasting insulin, (e) IPGTT, (f) IPITT, (g) total fasting cholesterol, (h) fasting triacylglycerides, (i) HOMA2‐%B and %S, and (j) HOMA2‐IR of the rats before and after 8 weeks of treatment. Error bars represent ± standard error of mean. *p < 0.05, **p < 0.01, and ***p < 0.001 relative to Lean and ^*p* < 0.05, ^^*p* < 0.01, and ^^^*p* < 0.001 relative to STZ

From Figure 1, it can be seen, that the different cannabis concentrations namely CE1.25, CE2.5, and CE5.0 had no significant effect on weight gain, weight gain per calorie intake, IPGTT (CE1.25 showed a slight improvement, although not significant), fasting triacylglycerides when compared to STZ‐untreated control. On the other hand, CE1.25 significantly (*p* < 0.01) improved fasting blood glucose levels, reduced fasting insulin levels, increased HOMA2‐%S (without affecting HOMA2‐%B) when compared to the STZ control, thereby potentially reversing the insulin‐resistant state of the rats. Higher cannabis concentrations as well as MET, had the opposite effect where HOMA‐2%B was significantly increased without affecting the HOMA2‐%S. Possibly a low cannabis concentration (CE1.25) being unable to promote or increase insulin secretion (HOMA‐%B) in STZ‐induced rats, compensated by rather improving insulin‐sensitivity (HOMA‐%S) of the peripheral tissues as a way to regulate glucose homeostasis. HOMA2‐IR is often used as a predictive marker of type 2 diabetes. Surprisingly, the Lean group exhibited a significantly higher HOMA2‐IR than the STZ‐induced rats indicating a more pronounced diabetic state. However, studies have shown that HOMA2‐IR (without compromised beta cell activity) on its own it cannot be used as a predictive marker for diabetes (Ghasemi et al., 2015; Khardori, 2013) and both HOMA2‐%B together with HOMA2‐%S should be reported. In this study, HOMA2‐%B of the Lean group was preserved when compared to the STZ‐induced rats, thereby indicating a healthy state. Of all the STZ‐induced rats with impaired beta cell activity, CE1.25 exposure interestingly was the only treatment able to restore the HOMA2‐IR of the rats hereby emphasizing the importance of optimum dosage.

Increasing cannabis concentrations improved the fasting total cholesterol levels of the rats. Metformin, on the other hand, was efficient in significantly improving fasting glucose levels, HOMA2‐%B and resulted in reduced weight gain (*p* < 0.05) when compared to the STZ‐untreated groups. This is in accordance with current literatures where the use of metformin has been shown to promote weight loss and is one of the most commonly, prescribed anti‐diabetic drugs amongst diabetic patients (Firouzjaei et al., 2016; Shurrab & Arafa, 2020). From Figure 1, the anti‐obesity properties of cannabis is questionable since no significant reduction in weight gain was observed relative to the STZ control. CE2.5 did cause less weight gain per calorie intake when compared to STZ, however, data were not significant. Possibly longer treatment period was required to observe noticeable change in weight and/or potentially metabolic healthy obesity was induced, restoring insulin‐sensitivity. It is therefore of great interest to determine the effect mediated by cannabis treatment especially at the various adipose tissue level, which plays a crucial role in energy metabolism and body weight.

### 3.2 The effect of the cannabis treatment on mitochondrial to genomic (MT:18S) DNA ratio of various fat depots

Adipose tissue is one of the main peripheral organs that plays a crucial role in glucose and lipid homeostasis (Czech, 2020; Stanford et al., 2013) and often adipose dysfunction can result in metabolic disorders such as obesity and insulin resistance (Ahmed et al., 2021). From Figure 1, it is evident that CE1.25 has potential therapeutic properties where insulin resistance is reversed. However, those parameters were measured *in vivo*. Since different fat depots in an organism play different roles (Hill et al., 2018; Maurer et al., 2021), it was difficult to determine, on which main fat depots, CE1.25 was potentially exerting its anti‐diabetic effect. To gain some insights, the MT:18S DNA in various fat deposits namely abdominal peritoneal, intramuscular, mesenteric, epididymal, brown, pancreatic, and sternum fat was determined.

**FIGURE 2:**
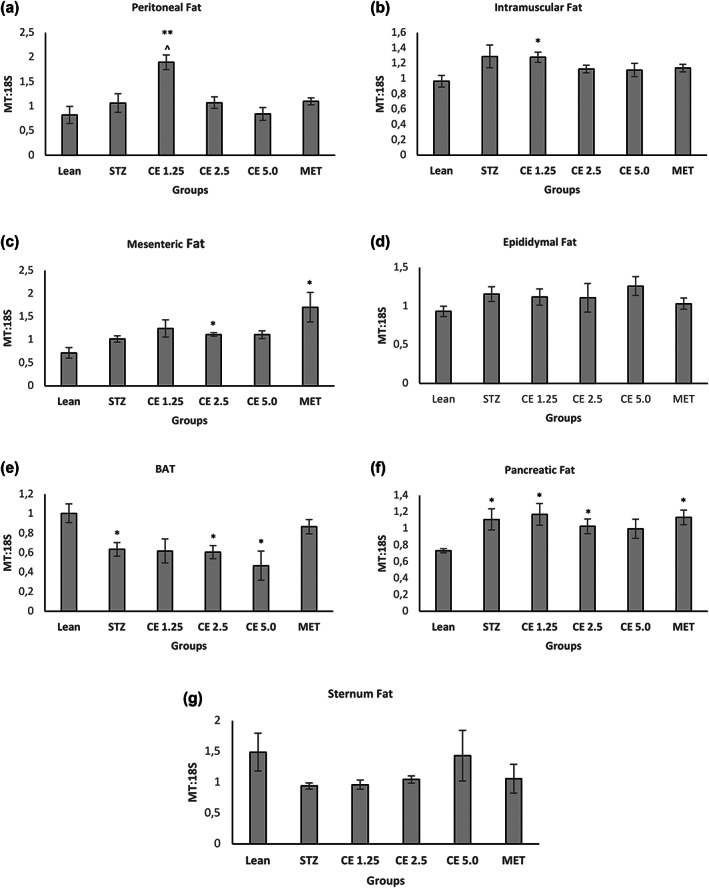
Mitochondrial to Genomic DNA ratio of various fat deposits. Mitochondrial to Genomic DNA ratio was determined across various fat deposits namely (a) Peritoneal, (b) Intramuscular, (c) Mesenteric, (d) Epididymal, (e) BAT, (f) Pancreatic, and (g) Sternum fat. Error bars represent ± standard error of mean (*n* = 6–7). *p < 0.05, **p < 0.01, ***p < 0.001 relative to Lean and ^*p* < 0.05 relative to STZ

From Figure 2, at the peritoneal level, only CE1.25 was able to significantly increase the MT:18S DNA when compared both to the STZ (*p* < 0.05) and Lean group (*p* < 0.01). There was also a significant (*p* < 0.05) increase in MT:18S DNA at the intramuscular level, however, only when compared to the Lean group and not to the STZ untreated control. CE2.5 and MET treatment caused a significant increase in MT:18S DNA in the mesenteric fat, relative to the Lean group. At the epididymal and sternum levels, the MT:18S DNA was fairly stable across all the groups. In BAT, interestingly only CE1.25 and MET were able to restore the MT:18S DNA fairly similar levels as the Lean group and lastly in the pancreatic fat, all the treatments except CE5.0 caused a significant increase in MT:18S DNA, relative to the Lean group. Only CE1.25 was able to significantly affect the MT:18S DNA across the different fat depots especially when compared to the STZ control. Interestingly, it has been reported that the mitochondrial copy number is not influenced by weight loss, or insulin sensitivity or energy expenditure but is strongly associated with lipogenesis in adipocytes (Kaaman et al., 2007). On the other hand, mitochondrial density has also been found to be reduced in adipocytes of diabetic rats (Christe et al., 2013) and the use of thiazolidinediones (TZDs) help to increase/restore mitochondrial copy number and increase fatty acid oxidation in diabetic patients (Bogacka et al., 2005). However, TZD's have also been reported to promote weight gain (Granberry et al., 2007). Therefore, it is unclear where an increase in mitochondrial copy number is directly involved in increased fatty acid oxidation or an increase in mitochondrial copy number is required to meet the energy demand for triacylglycerides synthesis, in order to protect other peripheral organs such as the liver, heart, and muscle from lipotoxicity. Further research is required to understand and unfold the role of mitochondrial copy number in adipose tissue.

### 3.3 Histological analysis of adipocytes of various fat depots

**FIGURE 3:**
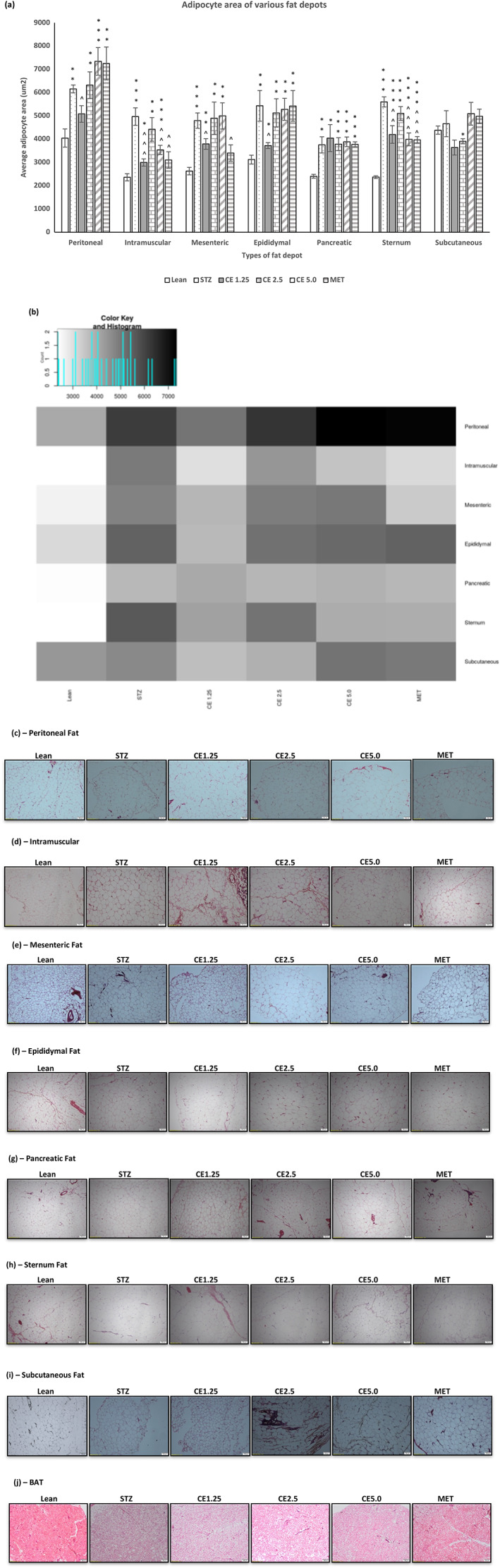
The morphological changes of the adipocytes of fat depots across different treatment groups. Figure depicting the average adipocytes area of (a) various fat depots. Error bars represent ± standard error of mean (*n* = 7). *p < 0.05, **p < 0.01, ***p < 0.001 and relative to Lean and ^*p* < 0.05, ^^*p* < 0.01 and ^^^*p* < 0.001 relative to STZ. A heatmap (b) was generated using the shiny heatmap software for easy visualization of data. The effect observed is dependent is not only dependent on the concentration of cannabis administered but also on the type of fat depot. Micrographs illustrating the difference in morphology of the adipocytes of (c) Peritoneal, (d) Intramuscular, (e) Mesenteric, (f) Epididymal, (g) Pancreatic, (h) Sternum, (i) Subcutaneous, and (j) BAT fat across the different treatment groups

Since it was unclear how MT:18S DNA and fat metabolism (oxidation or synthesis) was linked, the adipocytes area of various fat depots was measured across all the different treatment groups and the results are shown in Figure 3.

Surprisingly, CE1.25 was able to significantly reduce the average adipocyte area of peritoneal (*p* < 0.05), intramuscular (*p* < 0.0001), mesenteric (*p* < 0.05), epididymal (*p* < 0.05), and sternum (*p* < 0.01) fat when compared to the untreated STZ group but had no effect at the pancreatic and subcutaneous fat level (relative to STZ). As mentioned before, metformin is known for promoting weight loss in diabetic patients (Shurrab & Arafa, 2020) and based on the current findings, it can be hypothesized that its main mechanism of action might be at the intramuscular (*p* < 0.01), mesenteric (*p* < 0.05), and sternum (*p* < 0.001) fat level, where the average adipocyte area was significantly less than the untreated STZ group. Interestingly, CE2.5 had no significant effect on adipocyte area in all the fat depots investigated, whereas CE5.0 was able to significantly reduce the adipocyte area of intramuscular (*p* < 0.01) and sternum (*p* < 0.001) fat when compared to STZ group. From these results it is clear that the effect mediated by the different cannabis concentrations, is dependent on the type of fat depot and further research is required to address the following questions: Is the effect observed linked to the size and location of the adipose tissue depot? Expandability limit of the adipocytes? The number of cannabinoid receptors present on the adipocytes? Metabolic activity/flexibility of the fat depot? Such information will be of great value to understand the precise mechanism of action of cannabis in fat tissues. From Figures 2 and 3, the only positive correlation between MT:18S and adipocyte area observed was in peritoneal and intramuscular fat, where CE1.25 caused an increase in MT:18S DNA in both tissue type, which might account for the decrease in adipocyte area possibly due to increased fat metabolism. For this reason, further in‐depth research was conducted using the peritoneal and intramuscular fat as opposed to the other fat depots.

### 3.4 Mitochondrial activities of peritoneal and intramuscular fat

Abdominal peritoneal fat is one of the main fat depots which contribute to the development of insulin‐resistance and diabetes (Hsieh et al., 2014). Its ability to expand during lipid overload provides a protective mechanism to prevent lipotoxicity to other non‐adipose tissues. However, when the limit of expansion is exceeded, it results in lipid overflow which is detrimental to other organs and promotes the development of insulin‐resistance and diabetes (Virtue & Vidal‐Puig, 2010). Another fat depot playing an important role in diabetes, is the intramuscular fat, where it is closely linked to the occurrence of skeletal muscle insulin resistance (Therkelsen et al., 2013). Skeletal muscle is one of the main organs involved in glucose and lipid metabolism and a loss in insulin sensitivity is often associated with increasing lipid intermediates, due to a static pool of intramuscular fat, which displays a low metabolic activity (Shaw et al., 2010). It has been found to be one of the early markers for diabetes, preceding beta cell dysfunction (Kitessa & Abeywardena, 2016).

**FIGURE 4:**
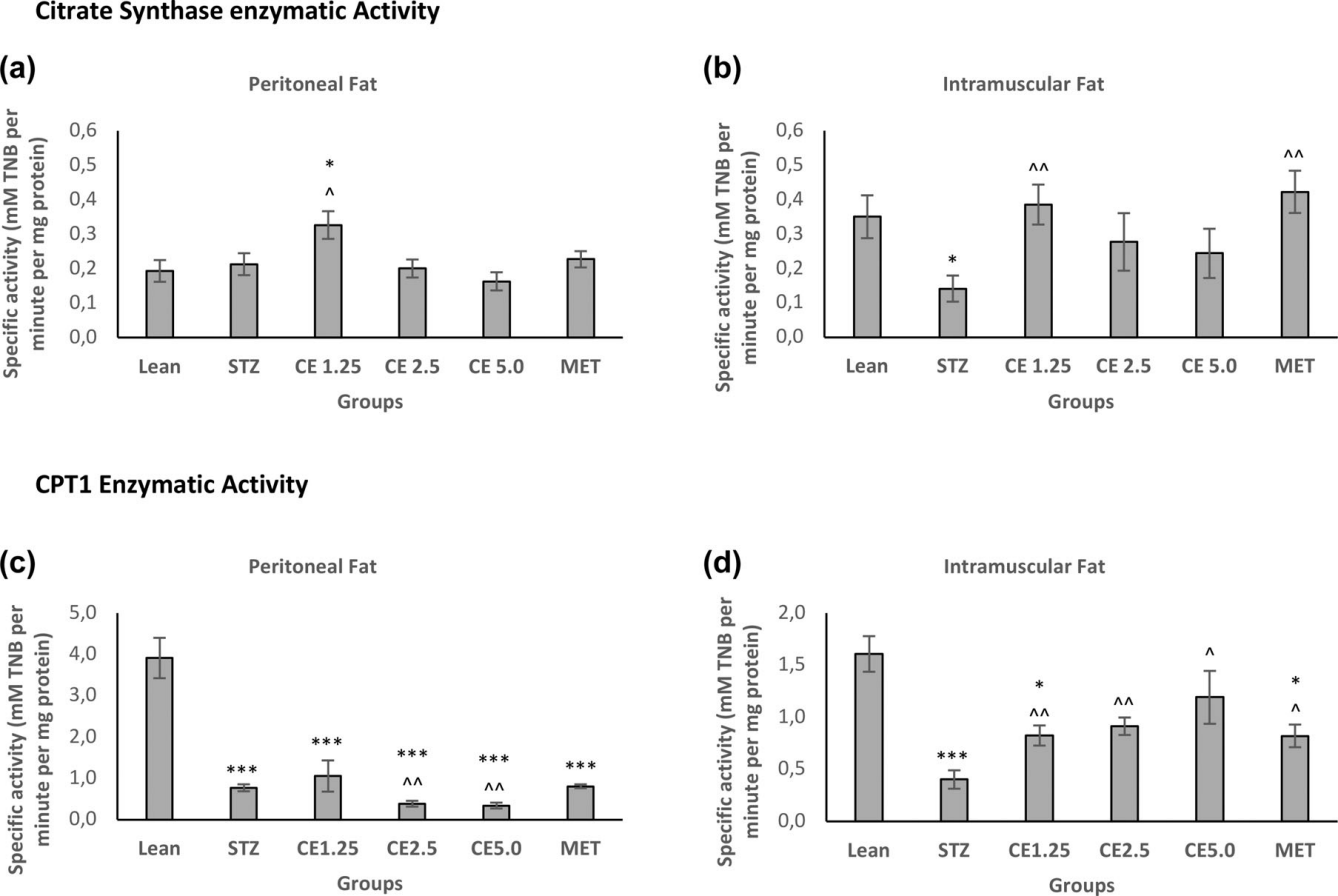
Citrate synthase and CPT1 activity in Peritoneal and Intramuscular Fat. Mitochondrial activity namely citrate synthase and CPT1 enzyme activities were measured both in Peritoneal (a,c) and Intramuscular Fat (b,d) respectively. Error bars represent ± standard error of mean (*n* = 6–7). *p < 0.05, **p < 0.01, ***p < 0.001 relative to Lean and ^*p* < 0.05, ^^*p* < 0.01 relative to STZ

The mitochondrial enzyme citrate synthase is one of the key enzymes of the tricarboxylic acid cycle (TCA) (Chen et al., 2014; Civitarese et al., 2007) and its activity in adipose tissue is reduced in cases of obesity and insulin‐resistance (Christe et al., 2013). From Figure 4, it can be seen that CE1.25 significantly increased citrate synthase activity relative to the untreated control both in peritoneal (*p* < 0.05) and intramuscular fat (*p* < 0.01) tissue. On the other hand, metformin was only efficient at the intramuscular fat, where the enzyme activity was significantly increased (*p* < 0.01) relative to the STZ control but not at the peritoneal fat level.

Such a finding is in accordance with published literature, where metformin has been shown to prevent skeletal muscle disorders by reducing intramuscular lipid deposition (Yang et al., 2020; Zabielski et al., 2017). It can therefore be hypothesized that the mechanism of action of CE1.25 in adipose tissue, might be at the mitochondrial level, where the enzyme of the TCA cycle is tightly regulated. It is, however, important to note that an increase in TCA cycle might also be due to increased ATP demand for TAG synthesis (Benador et al., 2018). As such, the enzyme activity of carnitine palmitoyl CoA transferase 1 (CPT1) which is the rate‐limiting step in fat oxidation (Schlaepfer & Joshi, 2020) and has been shown to boost mitochondrial activity and promote fat oxidation (Gong et al., 2019) was investigated. Interestingly, at the peritoneal level, CPT1 activity in all the STZ‐induced rats was unaffected when compared to the untreated control. This shows that fat oxidation was not influenced by the treatments, unlike the TCA cycle. The opposite was observed in the intramuscular fat. All the treatments significantly increased CPT1 activity, showing increased mitochondrial activity and fat oxidation when compared to the STZ group. It is evident that the different treatments affect the peritoneal and intramuscular fat deposits in a different way. The questions that now arise are: is it possible that (1) the peritoneal fat is mostly involved in lipogenesis as a protective mechanism to prevent lipid overflow (since the rats were fed a HFD) and have a different mechanism of mobilizing the fat droplets such as through beigeing and (2) the intramuscular fat displayed increased mitochondrial activity where beta‐oxidation is promoted due to reduced or no ability to promote beigeing? To help solve this puzzle, gene expression levels of markers involved in fat metabolism and fat beigeing was investigated.

### 3.5 Gene expression levels of selected markers across peritoneal, intramuscular and BAT fat depots

So far, it can be observed that CE1.25 had a positive/protective effect on fat metabolism across both peritoneal and intramuscular fat depots, however, the mechanism of action is fat depot‐dependent. One of the most common ways to reverse insulin resistance is to promote weight loss and healthy lifestyle specifically in obese and diabetic patients. Some studies have shown that cannabis administration helps to reduce weight gain (Levendal et al., 2012) and exhibit potential anti‐obesity properties (Ramlugon et al., 2018). In this study, however reduced weight gain was not observed, probably due to the preferred route of administration of the treatment (orally vs. intraperitoneally). Studies have shown that intra‐peritoneal administration of drugs in experimental animals, results in a more rapid and increased absorption of the drug as opposed to oral route of administration (Al Shoyaib et al., 2020). It is therefore hypothesized that a longer treatment period was required to observe significant weight loss but nonetheless from all the data thus far, it seems likely that a metabolic healthy obesity state was induced by the CE1.25 treatment.

**FIGURE 5:**
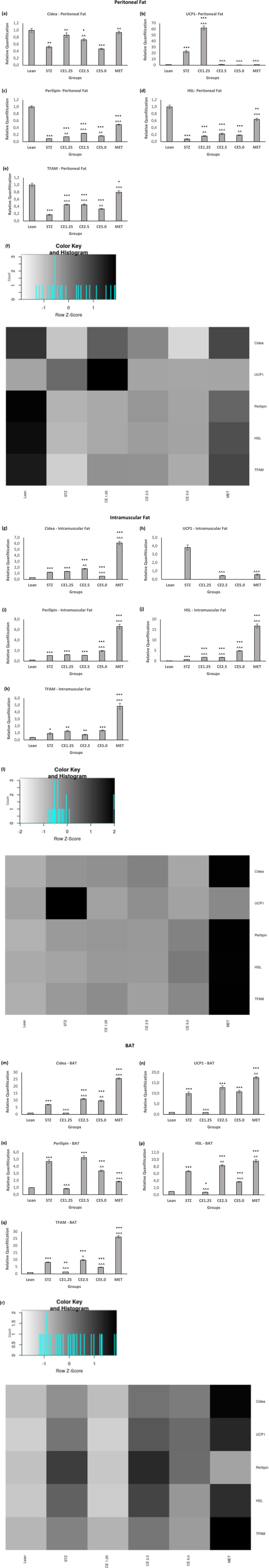
Gene expression levels of selected marker across Peritoneal, Intramuscular and BAT. The relative gene expression level of Cidea (a, g, m), UCP1 (b,h,n), Perilipin (c,i,o), HSL (d,j,p) and TFAM (e, k, q) across Peritoneal, Intramuscular, and BAT respectively. Error bars represent ± standard error of mean (*n* = 6–7). *p < 0.05, **p < 0.01, ***p < 0.001 relative to Lean and ^*p* < 0.05, ^^*p* < 0.01, ^^^*p* < 0.001 relative to STZ. Heatmaps for (f) peritoneal, (l) intramuscular and (q) BAT were generated using the shiny heatmap software. Light (white) block represents a low value while a dark box (black) represents a high value

The ability to promote fat beigeing is now considered as a therapeutic strategy to reduce weight gain or promote weight loss in obese individuals (Harms & Seale, 2013; Thyagarajan & Foster, 2017). To determine where CE1.25 and/or the other treatments, were promoting fat beigeing in peritoneal and/or intramuscular fat tissues, the gene expression level of selected markers involved in fat beigeing and fat metabolism namely, Cidea, UCP1, Perilipin, HSL, and TFAM were determined and compared to BAT fat tissue as the reference tissue (Figure 5).

Cidea is a beige fat marker (Kang et al., 2019; Lee et al., 2021). Interestingly, a low expression has been linked to a lean phenotype (Zhou et al., 2003), while a high expression has been linked to a metabolically healthy obese state by promoting expansion of adipocytes for lipid storage, thereby preventing lipotoxicity (Abreu‐Vieira et al., 2015). The second beige/brown fat marker investigated, namely UCP1, promotes thermogenesis (Bonet et al., 2017; Chouchani et al., 2019) and its level of gene expression is increased in cases of fat beigeing (Harms & Seale, 2013). HSL and perilipin, both are markers involved in fat metabolism, where perilipin is a protein associated with lipid droplets (Robenek et al., 2005; Wolins et al., 2006) and facilitate the breakdown of TAG by HSL (Girousse & Langin, 2012; Tansey et al., 2004), thereby regulating lipolysis in adipocytes. Lastly, TFAM, is a mitochondrial marker and plays an important role in mitochondrial biogenesis (Kozhukhar & Alexeyev, 2019; Picca & Lezza, 2015). Investigating the level of expression of the above‐mentioned markers, would assist in determining the potential mechanism of action of CE1.25 in peritoneal and intramuscular fat depots.

Figure 5 shows that in peritoneal fat, CE1.25 caused a significant increase in gene expression of the all the beigeing markers, when compared to the untreated STZ‐control. The same was observed in the case of MET (except for UCP1). In the intramuscular fat, CE1.25 only significantly increased HSL gene expression without affecting the other markers, whereas MET significantly increased the expression levels of all the markers investigated except for UCP1 relative to the STZ control group. This genes expression level results complement the CPT1 enzyme activity data, where at the peritoneal level, increased fat oxidation was not observed and instead it was hypothesized that a different mechanism of lipid mobilization was induced by CE1.25, in this case potentially through beigeing. At the intramuscular level, on the other hand, both an increase in mitochondrial beta‐oxidation as well as expression level of HSL (involved in fat metabolism) was observed and therefore in theory, beigeing is not metabolically “required”. Studies have shown that intramuscular fat consists of small lipids which are metabolically active only when there is regular depletion and renewal (for example in trained‐individuals), whereas in obese individuals with a sedentary lifestyle, the intramuscular fat depot is static, with enlarged adipocytes. This results in a downregulation of HSL, leading to the development of insulin‐resistance in the skeletal muscle (Shaw et al., 2010). Surprisingly in BAT, CE1.25 did the opposite to the gene activity profile observed in peritoneal fat. CE1.25 and MET treatments caused a significant decrease and increase (except perilipin) in all the gene markers investigated when compared to the STZ model respectively. Studies have shown that metformin exerts its anti‐obesity properties by inducing lipolysis in white adipose tissue to prevent fat accumulation and by enhancing the activity of brown fat where energy is dissipated as heat through UCP1 (Lv & Guo, 2020). This would explain  the increase in gene expression of fat metabolism markers namely perilipin, HSL and TFAM in the white adipose tissue (peritoneal and intramuscular fat) and increase in UCP1 gene expression levels in BAT. Furthermore, studies have shown that mice with impaired or reduced BAT activity, display increased beigeing of adipocytes in the white adipose tissue (Tan et al., 2015). Therefore, it is possible that the decrease in expression of these beigeing markers in BAT especially in the case of CE1.25, is linked or due to the increase in expression observed in peritoneal fat, which is a larger fat depot than BAT, as a way to maintain energy/heat balance or prevent energy wastage by BAT. Nonetheless, further studies need to be conducted to confirm all the findings.

## 4 CONCLUSION

Based on all the results of this study, the effect mediated by a cannabis extract vary in relation to the dose administered and the mechanism of action is dependent on the type of fat deposit. CE1.25 treatment was the only treatment that showed promising anti‐diabetic properties, where fasting blood glucose levels were significantly improved, the insulin‐resistant state of the rats was reversed and HOMA2‐%S of the rats were improved, as opposed to a higher concentration of cannabis treatment. Higher cannabis concentrations were ineffective in doing so, thereby emphasizing the biphasic nature of cannabis and the importance for an optimum dosage for specific therapeutic use. At the peritoneal level, CE1.25 increased citrate synthase enzyme activity, MT:18S DNA, expression levels of Cidea, UCP1, Perilipin, HSL and TFAM, resulting in reduced average adipocyte area. These findings suggest that CE1.25 might be promoting fat beigeing at the peritoneal level enabling the peritoneal fat to become metabolically more active and play a more efficient role in maintaining glucose and lipid homeostasis. At the intramuscular fat level, a different mechanism of action is observed. CE1.25 affects the mitochondrial activity, where an increase in gene expression level of HSL, citrate synthase and CPT1 (fat oxidation) enzyme activity is observed. The increase in mitochondrial activity and fat mobilization would explain the overall decrease in adipocyte area. Peritoneal adipocytes have greater ability to expand and accommodate fat droplets when compared to intramuscular fat. Hence, fat beigeing induced by the CE1.25 treatment at the peritoneal fat level might exert a protective role in the body by preventing lipotoxicity in non‐adipose tissues while increased mitochondrial activity in intramuscular adipocytes allows the adipocytes close to skeletal muscle to quickly mobilize the fat, thereby protecting the organs from lipid overload and insulin resistance.

## 5 LIMITATIONS OF THE STUDY AND FUTURE DIRECTIVES

The limitations of the study include the lack of (1) experiments involving the use of multiple cannabis extracts consisting of different cannabinoid ratios in order to evaluate the potential synergistic effects (2) experiments investigating the role of diet for example a high carbohydrate v/s high protein v/s a high fat diet in the effect mediated by oral cannabis administration (3) a time‐dependent study of oral cannabis administration on the insulin‐resistant and weight‐related parameters of the rats. Regarding the dose of cannabis administered, it should be noted that previous studies conducted have shown that the mean lethal oral dose (LD_50_) of THC in rats ranged from 800 to 1900 mg/kg depending on the strain and sex of the rats (Beaulieu, 2005). In this study, however, the concentration of THC present in the extract was much lower (a maximum THC concentration of 5 mg/kg was administered) and therefore the concentrations of cannabis extract was not toxic to the rats. Furthermore, due to time constrains, cost implications as well as ethical clearance issues, the above‐mentioned set of experiments could not be conducted and should be considered for future studies.

## CONFLICT OF INTEREST

The authors declare no conflict of interest.

## Supporting information


**Appendix S1.** Supporting Information

## Data Availability

Data would be made available on request.
