# Supplementary material for: Effect of oral cannabis administration on the fat depots of obese and streptozotocin‐induced diabetic rats
Source: Phytother Res. 2022 Nov 27;37(5):1806–22. doi: 10.1002/ptr.7694 (PMC10947483; doi:10.1002/ptr.7694)
Supplement: Supplementary file 1 — Appendix S1. Supporting Information [file PTR-37-1806-s001.docx]

**Supplementary information**


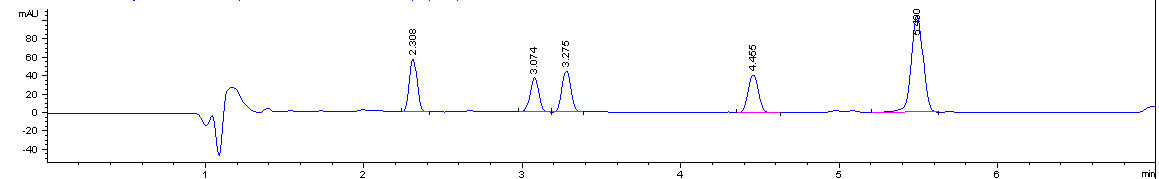


**CBDV**

**CBG**

**CBD**

**CBN**

**THC**

**Time (minutes)**

**Figure a: Typical chromatogram depicting the elution time and peak separation of the different standard cannabinoids in methanol mixed together.**

CBDV being the most polar relative to the other cannabinoids is eluted first followed by CBG, CBD, CBN and lastly THC (least polar) and they have retention times of 2.308, 3.074, 3.275, 4.455 and 5.490 minutes, respectively.


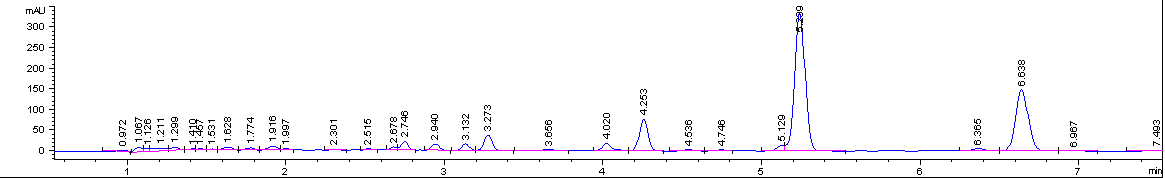


**THC**

**CBN**

**CBD**

**CBG**

**CBDV**

**Time (minutes)**

**Figure b: Typical chromatogram of the organic cannabis extract (in methanol), depicting the presence of the different standard cannabinoids in the extract.**
